# Whole-Body versus Local DXA-Scan for the Diagnosis of Osteoporosis in COPD Patients

**DOI:** 10.4061/2010/640878

**Published:** 2010-02-07

**Authors:** Lidwien Graat-Verboom, Martijn A. Spruit, Ben E. E. M. van den Borne, Frank W. J. M. Smeenk, Emiel F. M. Wouters

**Affiliations:** ^1^Department of Respiratory Medicine, University Medical Centre Maastricht, P. Debyelaan 25, 6229 HX Maastricht, The Netherlands; ^2^Department of Respiratory Medicine, Catharina Hospital Eindhoven, Michelangelolaan 2, 5623 EJ Eindhoven, The Netherlands; ^3^Research, Development & Education, CIRO, Centre of Expertise for Chronic Organ Failure, Hornerheide 1, 6085 NM Horn, The Netherlands

## Abstract

*Background*. Osteoporosis is an extrapulmonary effect of chronic obstructive pulmonary disease (COPD). Diagnosis of osteoporosis is based on BMD measured by DXA-scan. The best location for BMD measurement in COPD has not been determined. Aim of this study was to assess whole-body BMD and BMD of the hip and lumbar spine (local DXA) in COPD patients and compare the prevalence of osteoporosis at these locations. *Methods*. Whole body as well as local DXA-scan were made in 168 COPD patients entering pulmonary rehabilitation. Patient-relevant characteristics were assessed. Prevalence of osteoporosis was determined. Characteristics of patients without osteoporosis were compared to patients with osteoporosis on local DXA. *Results*. A higher prevalence of osteoporosis was found using local DXA compared to whole-body DXA (39% versus 21%). One quarter of patients without osteoporosis on whole body-DXA did have osteoporosis on local DXA. Significant differences in patient characteristics between patients without osteoporosis based on both DXA measurements and patients with osteoporosis based on local DXA only were found. *Conclusions*. DXA of the hip and lumbar spine should be made to assess bone mineral density in COPD patients. The lowest T-score of these locations should be used to diagnose osteoporosis.

## 1. Introduction

Chronic obstructive pulmonary disease (COPD) is characterized by a progressive airflow obstruction [1]. In addition, there are significant extrapulmonary effects, like osteoporosis. Osteoporosis is a systemic skeletal disease characterized by a low bone mineral density (BMD) and microarchitectural changes in bones leading to an increased bone fragility and, in turn, resulting in an increased fracture risk [2].

The prevalence of osteoporosis in COPD is higher than in healthy age-matched control subjects [3]. Moreover, COPD patients have a 60 to 70% higher risk of death following hip fracture than people without COPD [4]. It is therefore of high clinical importance to diagnose and treat osteoporosis in COPD according to international guidelines [2].

The gold standard for the diagnosis of osteoporosis is dual-energy absorptiometry (DXA) [2]. Multiple sites can be used to measure BMD by DXA. The sites most frequently used are the hip, the lumbar spine, forearm and/or whole-body. Several studies investigated the best location for DXA-scanning to diagnose osteoporosis [5–10]. DXA-scanning of the hip and the lumbar spine resulted in a higher prevalence of osteoporosis than whole-body DXA-scanning in pre- and postmenopausal women [6]. Indeed, the International Society for Clinical Densitometry advocates to measure BMD of the lumbar spine and the hip and to diagnose osteoporosis based on the lowest T-score of the measured locations [11]. To date, a comparison of BMD of various skeletal sites has not been performed in patients with clinically stable COPD. Therefore, the aim of this study was to compare the prevalence of osteoporosis using whole-body DXA-scanning versus local DXA-scanning (hip and lumbar spine) in male and female COPD patients. To identify risk factors for osteoporosis, patients without osteoporosis based on both DXA measurements were compared to patients with osteoporosis based on local DXA only.

## 2. Methods

The results of 168 consecutive COPD patients between November 2007 and May 2008 at CIRO, a centre of expertise for chronic organ failure in Horn (the Netherlands), were included in this retrospective study. All patients underwent all the tests during a 3-day baseline assessment before entering a patient-tailored pulmonary rehabilitation program [12]. Therefore, our manuscript does not include a statement of patient consent and the approval of Internal Review Boards.

Medical history, current medication use, and smoking status were assessed. Post-bronchodilator pulmonary function tests were performed (Jaeger MASTERLABBODY, VIASYS Healthcare) and COPD was diagnosed and classified according to the GOLD guidelines [1]. Body mass index (BMI) was defined as low (<21 kg/m^2^), normal (21–25 kg/m^2^), overweight (>25–30 kg/m^2^), and obese (>30 kg/m^2^). Fat-free mass index (FFMI) was assessed by bio-impedance analysis (BODYSTAT 1500 medical, single frequency; Xitron Technologies) and classified as depleted (men <16 kg/m^2^ and women <15 kg/m^2^) or normal [13]. A six-minute walking distance test was performed to estimate functional exercise capacity [14]. Arterial blood gases were collected to determine pH, arterial carbon dioxide tension (PaCO_2_), and arterial oxygen tension (PaO_2_).

Whole-body BMD as well as BMD at the femur and the lumbar spine (L1–L4) were assessed for each patient using a DXA-scan (Lunar Prodigy Ge-Lunar). Because the risk of hip and vertebral fractures is similar in men and women for any given BMD, we used the NHANES III data to calculate T-scores for both men and women [15, 16]. For whole-body DXA osteoporosis was defined by a T-score <−2.35, osteopenia: T-score between −2.35 and −0.9, and normal BMD: T-score >−0.9 [17]. For local DXA, osteoporosis was defined by a T-score of ≤−2.5, osteopenia as T-score between −1 and −2.5, and normal BMD: T-score >−1 [2]. To compare different locations of DXA-scan, we divided groups into osteoporosis and no osteoporosis (osteopenia and normal BMD combined) because only patients with osteoporosis need to be treated pharmacologically. 

### 2.1. Statistical Analyses

Discrete variables were compared with the Chi-square test and presented as percentages. Continuous variables were compared with independent *t*-test and presented as means ± standard deviation (SD). Pearson correlation was used. To estimate the relative risk a McNemars test was used. A *P*-value of <.05 was considered significant. All statistical analyses were performed using Statistical Package for Social Sciences (SPSS) version 16.0.

## 3. Results

Mostly male patients with clinically stable, mild to very severe COPD were studied. Based on whole-body DXA-scan prevalence of osteoporosis was 22.6% versus 38.7% based on hip and/or the lumbar spine DXA-scan (local DXA) (Table 1). 

Twenty-five percent of the 130 patients with a T-score >−2.35 as determined by whole-body DXA did have osteoporosis based on local DXA. In contrast, only 3.7% of the 103 patients with a T-score >−2.5 as determined by local DXA did have osteoporosis based on whole-body DXA (Table 2). This difference in prevalence was highly significant (*P* < .0001). The relative risk of having osteoporosis on local DXA in case of no osteoporosis on whole-body DXA was 6.4 times higher than the risk of having osteoporosis on whole-body DXA in case of no osteoporosis on local DXA (Table 2). 

Almost 16% of 122 patients with T-scores >−2.5 as determined by DXA at the lumbar spine did have osteoporosis based on DXA at the hip. In addition, a little over 18% of 126 patients with T-scores >−2.5 based on hip DXA-scanning did have osteoporosis on DXA-scan of the lumbar spine (Table 3). This difference was not significant (*P* = .644).

Significant differences were found in gender-distribution, proportion of GOLD IV patients, arterial blood gases, and functional exercise capacity between COPD patients without osteoporosis on whole-body DXA as well as local (hip and lumbar spine) DXA (group I) and those without osteoporosis on whole body DXA and with osteoporosis on local DXA (group II) (Table 4).

## 4. Discussion

Appreciating the possible impact of osteoporosis on fractures and survival in COPD [4], it is clinically important to have an early and accurate diagnosis. In the present study a method-specific difference in diagnosing osteoporosis has been found in patients with COPD. Indeed, whole-body DXA-scanning underestimates the prevalence of osteoporosis in COPD (Table 1). This finding is in accordance with the findings in pre- and postmenopausal women without COPD [6]. 

The prevalence of osteoporosis has been shown to differ when the same cut-off value for the T-score is used to define osteoporosis [18, 19]. This leads to an over- or underestimation of osteoporosis depending on the method used to asses BMD. In order to compare whole-body DXA to local DXA, we corrected for this difference in methodology by defining osteoporosis according to Boyanov [17] and the WHO [2], respectively. In turn, a good sensitivity-to-specificity ratio in the diagnosis of osteoporosis was obtained [17]. 

A possible explanation for the method-specific differences in prevalence of osteoporosis may be age-related. Indeed, loss of BMD of the lumbar spine occurs at a younger age than loss of BMD of the hip [6, 20]. In addition, due to degenerative changes of the spine at higher age, lumbar BMD could even be increased in the elderly. In the present trial the prevalence of osteoporosis was slightly higher using lumbar spine DXA (27.4%) compared to hip DXA (25.0%), while the prevalence of osteopenia was clearly higher in the latter group (33.3% versus 52.4%). Consequently, these patients may be at risk to develop osteoporosis. 

The International Society for Clinical Densitometry stated that the BMD of the spine and the hip should be measured and diagnosis of osteoporosis should be based on the lowest T-score of either the spine or the hip [11]. Indeed, in our COPD patients we found the prevalence of osteoporosis based on DXA of the hip to be 25%, of the lumbar spine 27.4%, and of the hip and lumbar spine combined 38.7% (Table 1). In combination with the fact that 25% of patients without osteoporosis on whole-body DXA had osteoporosis on local DXA, it seems reasonable to advise DXA of the hip and the lumbar spine and diagnose osteoporosis based on the lowest T-score in COPD patients. The costs of a whole-body DXA are the same as a local DXA scan. In addition, the costs of a DXA of the hip or the lumbar spine only are equal to the costs of a DXA scan of both the hip and lumbar spine. Therefore, there are no economical reasons to limit DXA-scan to the hip only. In addition, the time to make a local DXA scan is less than the time to make a whole body DXA. More research is needed to assess risk factors for osteoporosis in COPD in order to determine which patients should be evaluated for osteoporosis.

The current study showed that the group of patients without osteoporosis on whole-body DXA but with osteoporosis on local DXA consisted of significantly more females than males, patients with a lower six minutes walking distance and more patients with GOLD IV and higher PaCO_2_ and lower PaO_2_ levels as compared to patients without osteoporosis on both whole body- and local DXA (Table 4). Therefore, in this group of patients, it seems even more important to use local DXA to determine BMD instead of whole-body DXA. Even more because in the general population the fracture risk is increased in females [21] and in COPD patients, a higher prevalence of osteoporosis has been found in patients with higher GOLD classification and/or hypercapnic patients [22–26]. In the current study, the increased number of patients with GOLD IV COPD in group II is probably not due to increased airway obstruction (FEV1 was not significantly different in both groups), but to hypercapnia and/or hypoxemia (Table 4). Indeed, Dimai and colleagues found a significantly lower BMD and increased serum cross-linked telopeptide of type I collagen (a bone resorption marker) in hypercapnic patients suggesting that hypercapnia induces increased bone resorption [23]. With decreased daily physical activity, hip BMD especially will be decreased, which will be compensated by the BMD of other non-weight baring body parts when whole body BMD is assessed. 

When results of whole body DXA-scanning were added to local DXA-scanning, only 5 extra patients without osteoporosis on local DXA had a T-score of <−2.35 on the whole body DXA-scan (2.4%). 

To conclude, in future COPD trials, in which the diagnosis and/or treatment of osteoporosis is a primary outcome measure and in daily clinical practice, the most sensitive procedure for diagnosing osteoporosis should be used. On the basis of our findings, we advise to assess the BMD at the hip and lumbar spine and finally take the lowest T-score of these 2 locations to determine the prevalence of osteoporosis.

## Figures and Tables

**Table 1:** Patients characteristics.

	Total group (*N* = 168)	Men (*N* = 103)	Women (*N* = 65)	*P*-value
Age, years	63.6 ± 9.1 (39–85)	65.9 ± 8.6	60.0 ± 8.7	<**.001**
FEV1, %predicted	44.3 ± 18.5	45.06 ± 18.1	43.12 ± 19.2	.0510
GOLD I, %	6.0	6.8	4.6	.402
GOLD II, %	24.6	24.3	26.2	
GOLD III, %	26.3	30.1	20.0	
GOLD IV, %	43.1	38.8	49.2	

Arterial blood gas				
pH	7.43 ± 0.03	7.44 ± 0.03	7.43 ± 0.03	.078
PaCO_2_, kPa	5.4 ± 0.8	5.4 ± 0.9	5.6 ± 0.7	.106
PaO_2_, kPa	9.5 ± 1.4	9.5 ± 1.5	9.4 ± 1.3	.466
SO_2_, %	95 ± 3	94 ± 3	95 ± 2	.536

Smoking				
Ex-Smoker*, %	66.7	71.8	58.5	.073
Pack Years	38.8 ± 15.5	38.9 ± 15.0	38.8 ± 1603	.960
BMI, kg/m^2^	24.7 ± 4.4	24.7 ± 4.4	24.7 ± 4.5	.960
Low, %	20.8	21.4	20.0	.561
Normal, %	30.4	33.0	26.2	
High, %	38.1	34.0	44.6	
Obese, %	10.7	11.7	9.2	
FFMI, kg/m^2^	16.1 ± 2.1	16.8 ± 2.0	15.1 ± 1.8	<**.001**
Normal/Low, %	58.9/41.1	61.2/38.8	55.4/44.6	.458

Whole body DXA				
BMD, g/cm^2^	1.079 ± 0.118	1.113 ± 0.114	1.025 ± 0.103	<**.001**
T-score	−1.3 ± 1.4	−1.3 ± 1.4	−1.3 ± 1.3	.762
Normal BMD, %	40.5	41.7	38.5	.075
Osteopenia, %	36.9	31.1	46.2	.217
Osteoporosis, %	22.6	27.2	15.4	.081

DXA hip				
BMD, g/cm^2^	0.827 ± 0.149	0.858 ± 0.146	0.779 ± 0.143	**.001**
T-score	−1.7 ± 1.1	−1.6 ± 1.1	−1.9 ± 1.0	.151
Normal BMD, %	22.6	25.2	18.5	.400
Osteopenia, %	52.4	53.4	52.3	
Osteoporosis, %	25.0	21.4	29.2	

DXA LS				
BMD, g/cm^2^	1.065 ± 0.215	1.088 ± 0.211	1.027 ± 0.218	.074
T-score	−1.3 ± 1.8	−1.2 ± 1.8	−1.4 ± 1.8	.471
Normal BMD, %	39.3	44.7	30.8	.198
Osteopenia, %	33.3	30.1	38.5	
Osteoporosis, %	27.4	25.2	30.8	

DXA hip and LS				
Normal BMD, %	15.5	19.4	10.8	.175
Osteopenia, %	45.8	46.6	43.1	
Osteoporosis, %	38.7	34.0	46.2	
6MWD, m (*n* = 165)	420.61 ± 135.66	454.41 ± 132.57	368.62 ± 124.28	<**.001**
Oral corticosteroids, %	17,9	18.4	16.9	.802
Daily dose^#^, mg	5.9 ± 2.1 (2.5–10)	6.1 ± 2.1	5.7 ± 2.3	.653
Inhaled	85.7%	83.5	89.2	.301
corticosteroids, %				

Results are presented as means ± standard deviation, unless otherwise indicated.

*Rest of the patients are current smokers.

^#^Only patients using oral corticosteroids on a daily base (*n* = 30).

Abbreviations: FEV1 = forced expiratory volume in the first second; GOLD = Global Strategy for the Diagnosis, Management and prevention of COPD; COPD = chronic obstructive pulmonary disease; BMI = body mass index; FFMI = fat free mass index; DXA = dual energy absorptiometry; BMD = bone mineral density; LS = lumbar spine; 6MWD = six minutes walking distance; m = meters; PaCO_2_ = arterial carbon dioxide tension; PaO_2_ = Arterial oxygen tension; SO_2_ = Oxygen saturation.

**Table 2:** Osteoporosis versus no osteoporosis based on local DXA and whole body DXA.

Whole body DXA	Local DXA	
	Osteoporosis	No osteoporosis
Osteoporosis	33	5
No osteoporosis	32	98

Post Hoc test (McNemar): Relative risk 6.4; 95% confidence interval 2.93–32.06; *P* < .0001. (Relative risk: the risk of having osteoporosis on local DXA in case of a normal whole-body DXA is 6.4 times higher than the risk of having osteoporosis on whole body DXA in case of a normal local DXA.)

**Table 3:** Osteoporosis versus no osteoporosis based on DXA of the lumbar spine and of the hip.

DXA Hip	DXA LS	
	Osteoporosis	No osteoporosis
Osteoporosis	23	19
No osteoporosis	23	103

Abbreviations: LS = lumbar spine.

Post hoc test (McNemar): Relative risk 1.21; *P* = .644. (Relative risk: the risk of having osteoporosis on lumbar spine DXA in case of a normal hip DXA is 1.21 times higher than the risk of having osteoporosis on hip DXA in case of a normal lumbar spine DXA.)

**Table 4:** Differences between patients without osteoporosis and osteoporosis on local DXA.

	Group I (*N* = 98)	Group II (*N* = 32)	*P*-value
Male/Female, %	64/36	38/62	**.008**
Age, years	64.0 ± 9.2	61.7 ± 9.2	.229
FEV1, % predicted	46.6 ± 17.3	40.7 ± 20.9	.110
GOLD I, %	5	6	.803
GOLD II, %	29	19	.272
GOLD III, %	31	16	.097
GOLD IV, %	36	59	**.018**

Arterial blood gas			
pH	7.43 ± 0.03	7.42 ± 0.03	.267
PaCO_2_ (kPa)	5.38 ± 0.72	5.71 ± 0.85	**.033**
PaO_2_ (kPa)	9.62 ± 1.28	9.00 ± 1.51	**.024**
SO_2_ (%)	94.91 ± 2.54	93.73 ± 3.03	**.032**
BMI	25.88 ± 3.93	25.11 ± 4.03	.342
Low BMI, %	8	19	.093
Normal BMI, %	35	25	.309
High BMI, %	43	47	.691
Obese BMI, %	14	9	.474
6MWD, meters	443 ± 128	354 ± 146	**.002**

Group I = Patients without osteoporosis on total body DXA-scan and without osteoporosis on local DXA-scan.

Group II = Patients without osteoporosis on total body DXA-scan and with osteoporosis on local DXA-scan.

Results are presented as mean ± standard deviation or as % of the group.

Abbreviations: FEV1 = forced vital capacity in the first second; GOLD = Global Strategy for the Diagnosis, Management and prevention of COPD; COPD = chronic obstructive pulmonary disease; PaCO_2_ = arterial blood carbon dioxide tension; PaO_2_ = arterial blood oxygen tension; SO_2_ = oxygen saturation in arterial blood; BMI = body mass index. 6MWD = 6 minutes walking distance.
